# Ecology of fear in highly invasive fish revealed by robots

**DOI:** 10.1016/j.isci.2021.103529

**Published:** 2021-12-16

**Authors:** Giovanni Polverino, Vrishin R. Soman, Mert Karakaya, Clelia Gasparini, Jonathan P. Evans, Maurizio Porfiri

**Affiliations:** 1 Centre for Evolutionary Biology, School of Biological Sciences, University of Western Australia, Perth, WA 6009, Australia; 2 Department of Mechanical and Aerospace Engineering, Tandon School of Engineering, New York University, Brooklyn, NY 11201, USA; 3 Department of Biology, University of Padova, Padova, Italy; 4 Department of Biomedical Engineering, Tandon School of Engineering, New York University, Brooklyn, NY 11201, USA; 5 Center for Urban Science and Progress, Tandon School of Engineering, New York University, Brooklyn, NY 11201, USA

**Keywords:** Biological Sciences, Ecology, Zoology

## Abstract

Invasive species threaten biodiversity and ecosystem functioning. We develop an innovative experimental approach, integrating biologically inspired robotics, time-series analysis, and computer vision, to build a detailed profile of the effects of non-lethal stress on the ecology and evolution of mosquitofish (*Gambusia holbrooki*)—a global pest. We reveal that brief exposures to a robotic predator alter mosquitofish behavior, increasing fear and stress responses, and mitigate the impact of mosquitofish on native tadpoles (*Litoria moorei*) in a cause-and-effect fashion. Effects of predation risk from the robot carry over to routine activity and feeding rate of mosquitofish weeks after exposure, resulting in weight loss, variation in body shape, and reduction in the fertility of both sexes—impairing survival, reproduction, and ecological success. We capitalize on evolved responses of mosquitofish to reduce predation risk—neglected in biological control practices—and provide scientific foundations for widespread use of state-of-the-art robotics in ecology and evolution research.

## Highlights

• Can robotic predators reveal the vulnerabilities of invasive and pest species?
• Our predator selectively targets invasive fish to protect native amphibians
• Stress from the robot compromises behavior, health, and reproduction of invaders
• We open new frontiers for robotics in ecology, evolution, and biocontrol research

## Graphical abstract

**Figure:**
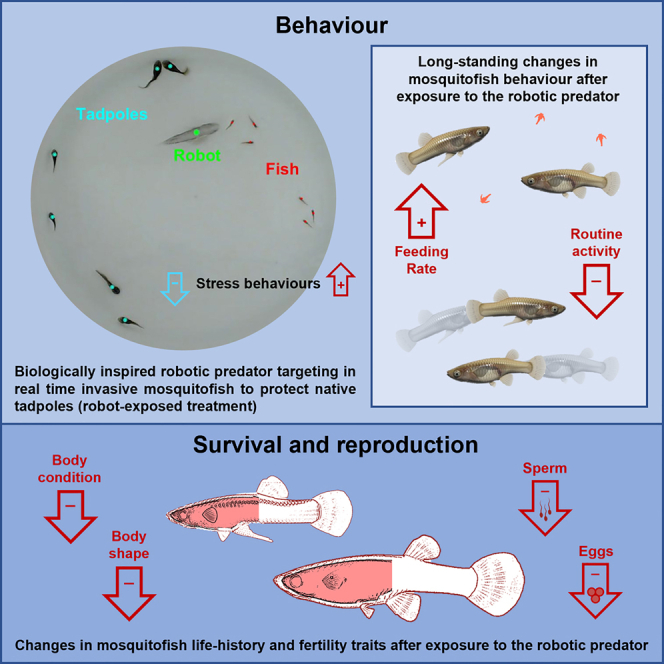

## Introduction

Human activities have resulted in the rapid redistribution of the world's biota, assisting some species to colonize regions far beyond their natural range (Elton, 2000; Richardson, 2011). The consequences of such invasions have been severe, with invasive species disrupting ecological communities (Lockwood, 2013), driving population declines and species extinctions (Bellard et al., 2016; Clavero and García-Berthou, 2005), and costing billions of dollars globally every year (Diagne et al., 2021). Despite these longstanding ecological and economic impacts, we generally lack effective control measures for mitigating the spread of invasive species, which remains an urgent environmental challenge (Byers et al., 2002; Su et al., 2021).

A highly promising, but under-developed, approach to counter animal invasions is to alter the behavior of invaders, especially ecologically relevant behaviors that can impact their fitness and invasion ability (Culshaw-Maurer et al., 2020). Growing evidence suggests that non-lethal stress—especially fear from perceived predation risk (Lima, 1998a; Peacor et al., 2020)—has substantial and negative effects on the behavior and ecology of animals (Killen et al., 2013). In fact, predation risk requires animals to adjust their behavior so that they are more difficult to capture, detect, or encounter (Lima, 1998a). As a result, predators have large impacts on ecological systems, independent of actual predation. For instance, effects of non-lethal stress from predation risk can range from changes in the physiology of prey animals to alterations of ecosystems functioning (Hawlena and Schmitz, 2010; Lima, 1998b).

Because the whole scale killing of invaders is likely to be impractical and ethically questionable, developing fundamental knowledge from predator-prey ecology for informing a new generation of biocontrol practices becomes of paramount importance. However, until recently technological and conceptual gaps have prevented the pursuit of this research direction. Precise measurements of the ecological implications of non-lethal stress have been hard to obtain (Lima, 1998a), and their use to inform biological control practices is uncharted.

**Figure 1:**
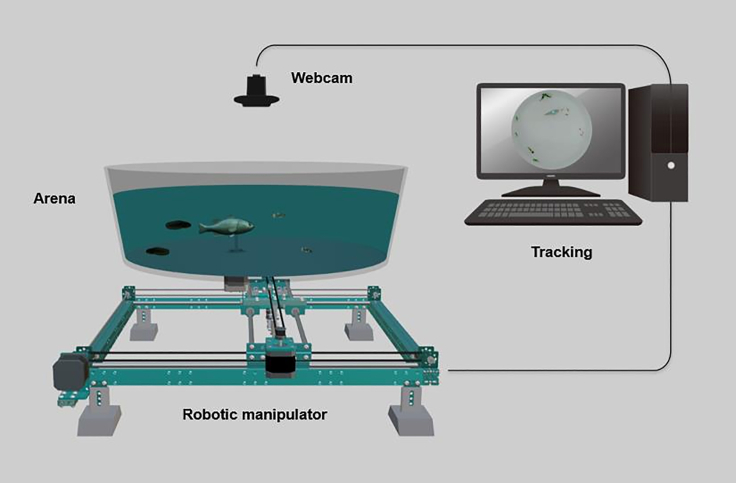
Schematic of the robotic platform used for exposing mixed groups of mosquitofish and tadpoles to the biologically inspired, interactive robotic predator The high-precision robotic manipulator was positioned below the experimental arena in which mosquitofish, tadpoles, and the robotic predator were located. The webcam was installed above the arena to acquire the video feed for the multi-species tracking system that was used to quantify interactions between the robotic predator and live animals in real time. In the figure, we present the arena as transparent to offer a better view of the apparatus—in practice, the walls of the experimental arena were white and opaque.

Here, we explore the potential of biologically inspired, interactive robotic predators to selectively alter the behavior of eastern mosquitofish (*Gambusia holbrooki*; Figure 1)—a model species in ecology and evolution, and one of the world's worst invasive species (Lowe et al., 2000) that threatens freshwater fishes and amphibians (Komak and Crossland, 2000; Pyke, 2008). We developed a robotic predator that closely mimics the appearance and movement patterns of the mosquitofish's main native predator—the largemouth bass (*Micropterus salmoides;* García-Berthou, 2002; Wicker and Johnson, 1987). A computer-vision system allowed the robot to differentiate in real time between mosquitofish and tadpoles of a common native Australian frog (*Litoria moorei*), which is negatively impacted by mosquitofish in the wild (Reynolds, 2009). So the robot simulated realistic attacks toward mosquitofish (Polverino et al., 2019) when they approached tadpoles (Figure 1). To detail the cause-and-effect relationships underlying interactions between mosquitofish, tadpoles, and the robotic predator, we conducted a model-free information-theoretic analysis (Neri et al., 2017). We combined evolutionary approaches with ecological perspectives to uncover the impacts of non-lethal exposure to the robotic predator on mosquitofish behavior, life history, and reproductive traits.

We first established 12 independent mesocosm tanks, where we randomly housed six wild-caught mosquitofish and six wild-caught tadpoles per tank. We assayed the group behavior of mosquitofish and tadpoles twice a week for seven consecutive weeks, transferring each mixed group of mosquitofish and tadpoles from their mesocosm tank into the experimental arena (Figure 1) for 1h per trial (168 trials). In the first week, all mixed groups of mosquitofish and tadpoles were assayed in the absence of the robotic predator, to test for their baseline behavior. During the following five weeks, groups were either exposed (treatment) or non-exposed (control) to the robotic predator, assaying their group responses in either the presence or absence of the robotic predator. All groups were assayed again in the absence of the robotic predator on the last week. Over the seven weeks, we also monitored the routine activity and feeding rate of mosquitofish in their housing mesocosm tanks twice a day for four days a week (672 trials)—routine activity (5 min) and feeding rate (1 min) per individual—following standard protocols (Polverino et al., 2018; Schröder et al., 2016). We measured body size, weight, and shape of each mosquitofish before the study started and after its conclusion. From these data, we calculated the body condition of all individuals at both time points as a proxy for their energy reserves. At the end of the experiment, we measured sperm traits (sperm number and quality) in males and number and weight of eggs in female mosquitofish to infer fertility. This design allowed us to test whether effects from the exposure to the robotic predator extended beyond the experimental arena, and exerted long-term impacts on the behavior, life history, and fertility of mosquitofish.

We expected that mosquitofish would adjust their behavior to minimize predation risk during exposure to robotic predators (Polverino et al., 2019; Polverino and Porfiri, 2013a; b), thereby favoring escape strategies of native tadpoles. We also anticipated that the non-lethal stress associated with brief exposures to the robotic predator might cause long-lasting changes in the behavioral and life-history strategies of mosquitofish, with individuals investing in survival over reproduction (Ydenberg and Dill, 1986). Indeed, behavioral adjustments have been shown previously to play a critical role in mosquitofish ecological success, determining growth and survival (Brodin et al., 2019; Polverino et al., 2016), and fitness outcomes (Polverino et al., 2018). Compared to individuals not exposed to predation risk, we hypothesized that fish exposed to the robotic predator would exhibit lower routine activity and feeding rates even weeks after the exposure, along with lower body condition and fertility. In contrast, we did not expect the robot to affect the behavior of tadpoles. Tadpoles are not only less sensitive to visual stimuli than mosquitofish (vS Hoff et al., 1999; Ward and Mehner, 2010), but they are also evolutionarily naive to the predator species which inspired the design of the robot (Cox and Lima, 2006), and are not chased by the interactive robot.

## Results

### Acute effects: group-behaviour measures

**Figure 2:**
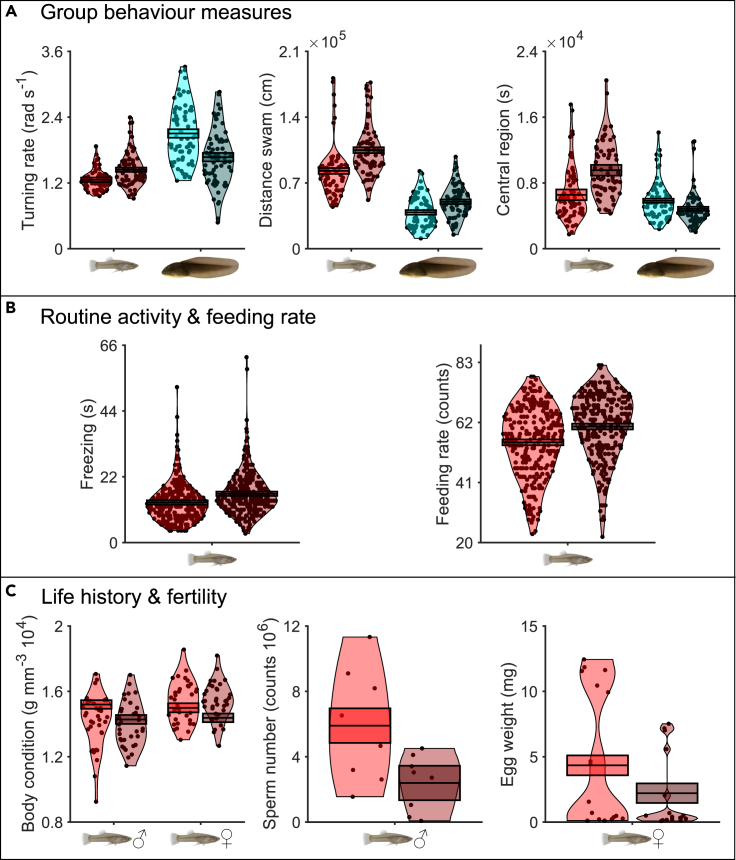
Violin plots depicting significant variation in behavior, life-history, and fertility traits between non-exposed (light-coloured violins) and robot-exposed animals (dark-coloured violins) (A) Group-behaviour measures of mosquitofish and tadpoles in the experimental arena, (B) routine activity and feeding rate of mosquitofish in their housing mesocosm tanks, and (C) life-history and fertility traits of mosquitofish weeks after behavioral assays in the experimental arena. Raw data (violins) and estimated marginal means (EMMs, boxes) are presented. EMMs allow refined comparisons between treatments on each trait after accounting for the contribution of covariates (fixed and random effects) included in the model. Plots are presented only for traits in which the fixed effect treatment explained a significant portion of the variation observed in the model.

Our results confirm that a biologically inspired, interactive robotic predator alters the behavioral dynamics of mosquitofish and tadpoles when mixed together. We found substantial differences in sociality, activity, and space use between mixed groups of mosquitofish and tadpoles exposed (treatment) and non-exposed (control) to the robotic predator (Figure 2A and Tables 1 and S1).

**Table 1:** Results from the fixed-factor structure of the linear-mixed models on group-behaviour measures

Model	Mosquitofish				Tadpoles				Mosquitofish & tadpoles			
	Mean sq.	*df*	*F*	*p*	Mean sq.	*df*	*F*	*p*	Mean sq.	*df*	*F*	*p*
**AFND (cm)**												

Treatment	0.258	1,10	0.048	0.830	8.505	1,10	4.094	0.071	1.900 10^1^	1,10	7.224	**0.023**
Week	2.909 10^1^	5,121	5.436	**<0.001**	6.492	5,121	3.125	**0.011**	2.223 10^1^	5,121	8.450	**<0.001**
Trial	1.848 10^1^	1,121	3.455	0.065	3.951	1,121	1.902	0.170	2.313 10^1^	1,121	8.792	**0.004**
Treatment × week	1.254 10^1^	5,121	2.344	**0.045**	4.424	5,121	2.129	0.066	2.595	5,121	0.986	0.429

**AIID (cm)**												

Treatment	0.701	1,10	0.283	0.606	1.599	1,10	1.531	0.244	5.141	1,10	3.628	0.086
Week	9.193	5,121	3.718	**0.004**	3.800	5,121	3.639	**0.004**	7.470	5,121	5.272	**<0.001**
Trial	4.722	1,121	1.910	0.169	0.955	1,121	0.914	0.341	8.819	1,121	6.223	**0.014**
Treatment × week	6.578	5,121	2.661	**0.026**	2.105	5,121	2.016	0.081	1.972	5,121	1.392	0.232

**Turning rate (rad s^−1^)**												

Treatment	0.455	1,10	1.069 10^1^	**0.008**	3.829	1,10	1.558 10^1^	**0.003**	–	–	–	–
Week	0.310	5,121	7.289	**<0.001**	0.527	5,121	2.146	0.064	–	–	–	–
Trial	0.096	1,121	2.267	0.135	0.080	1,121	0.326	0.569	–	–	–	–
Treatment × week	0.246	5,121	5.853	**<0.001**	0.466	5,121	1.895	0.100	–	–	–	–

**Distance swam (cm)**												

Treatment	1.209 10^10^	1,10	2.525 10^1^	**<0.001**	2.666 10^9^	1,10	1.098 10^1^	**0.008**	–	–	–	–
Week	7.591 10^9^	5,121	1.585 10^1^	**<0.001**	9.236 10^8^	5,121	3.804	**0.003**	–	–	–	–
Trial	1.818 10^9^	1,121	3.796	0.054	2.230 10^6^	1,121	0.009	0.924	–	–	–	–
Treatment × week	4.056 10^8^	5,121	0.847	0.519	2.494 10^8^	5,121	1.027	0.405	–	–	–	–

**Time in central region (s)**												

Treatment	7.350 10^7^	1,10	1.045 10^1^	**0.009**	3.497 10^7^	1,10	7.982	**0.005**	–	–	–	–
Week	5.910 10^7^	5,121	8.406	**<0.001**	1.142 10^7^	5,121	2.606	**0.028**	–	–	–	–
Trial	5.902 10^7^	1,121	8.395	**0.004**	2.348 10^7^	1,121	5.359	**0.002**	–	–	–	–
Treatment × week	8.309 10^6^	5,121	1.182	0.322	6.932 10^6^	5,121	1.582	0.169	–	–	–	–

The dependent variables, social cohesion (average furthest neighbor distance, AFND, and average inter-individual distance, AIID), activity (turning rate and distance swam), and space use (time in the central region), are tested separately. Treatment (non-exposed and robot-exposed), week (two to seven), interaction (treatment × week), and trial (two repeated measures per tank per week) are included as fixed effects in each model. Random intercepts are also included for each mesocosm tank in each model, which allowed accounting for repeated measures. Analysis of variance was performed with Satterthwaite's method. Significant results are in bold.

In particular, social cohesion increased from non-exposed to predator-exposed fish, with lower inter-individual distances between group members in the presence of the robot. Both the average distance between a mosquitofish and its furthest neighbor (average further neighbor distance, AFND) and the average distance between all mosquitofish (average inter-individual distance, AIID) were lower in robot-exposed than non-exposed groups, especially over the first few weeks of exposure (Table 1). By contrast, the presence of the robotic predator had no impact on the social cohesion of tadpoles (Table 1).

We found opposing swimming (activity) patterns in mosquitofish and tadpoles in response to the robotic predator. Mosquitofish exhibited elevated levels of erratic swimming (high turning rate) in the robot-exposed compared to the non-exposed treatment, whereas tadpoles showed the opposite pattern (Figure 2A, left panel). Yet, both mosquitofish and tadpoles swam further distances when exposed to the robotic predator (Figure 2A, central panel, and Table 1).

The robotic predator altered the space use of mosquitofish and tadpoles in opposite ways. Mosquitofish and tadpoles spent more and less time, respectively, in the central region of the arena when exposed to the robot (Figure 2A, right panel, and Table 1).

**Figure 3:**
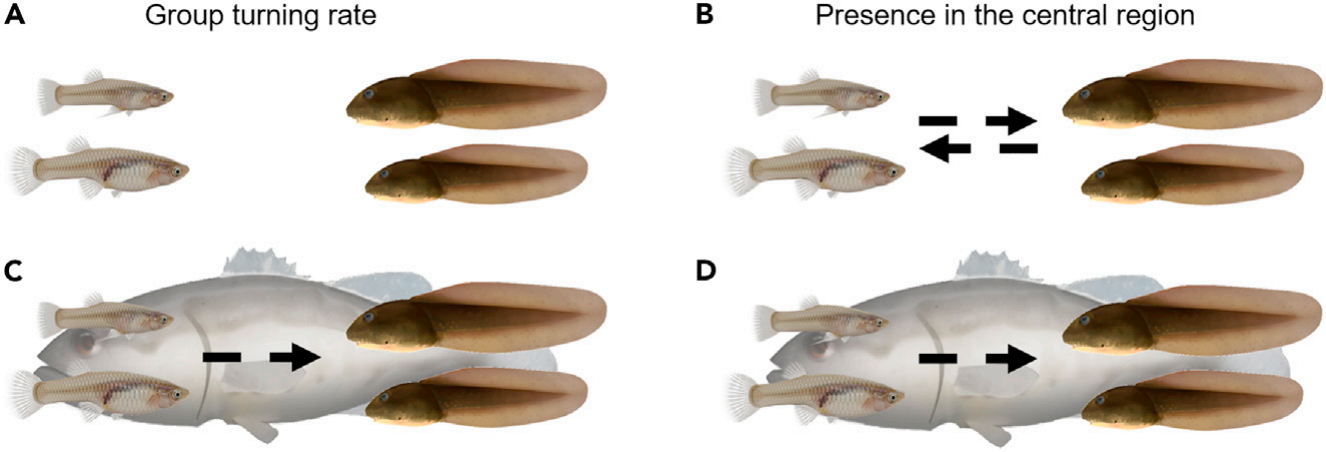
Graphical representation of the transfer entropy between mosquitofish and tadpoles in absence (non-exposed, panels A and B) and presence (robot-exposed, panels C and D) of the biologically inspired, interactive robotic predator. Transfer entropy measures the uncertainty reduction in predicting the future behavior of one species from its present because of additional knowledge about the present behavior of the other species. Transfer entropy is computed on a symbolic time series of the average magnitude of group turning rate (panels (A) and (C)) and presence in the central region of the arena (panels (B) and (D)). Dashed arrows represent significant information transfer (entropy) from one species to the other.

Through the use of transfer entropy on experimental time series (Porfiri, 2018), we examined cause-and-effect relationships underlying the observed changes in behavioral patterns of each species (Figure 3 and Table S2). The robot favored the unidirectional interaction between tadpoles and mosquitofish, such that the turning rate of tadpoles was influenced by mosquitofish, but not *vice versa* (Figures 3A and 3C); tadpoles did benefit from the increased stress in mosquitofish associated with the presence of the robot. Transfer entropy analyses also clarified the role of the robotic predator on the differential space use of the two species. The space use of mosquitofish and tadpoles was mutually influenced in the absence of the robot (non-exposed treatment; Figure 3B); when the robotic predator was present, the mutual influence disappeared in favor of a unidirectional interaction with only tadpoles responding to mosquitofish (Figure 3D and Table S2). Thus, the robotic predator was effective in spatially separating the two species, inhibiting mosquitofish from chasing tadpoles, and allowing tadpoles to distance from mosquitofish.

### Routine activity and feeding rate

Brief exposures to the robotic predator had long-term consequences for mosquitofish behavior (Figure 2B, Tables 2 and S3). Individuals exposed to the robot were less active and displayed more anxious behaviors when in their housing mesocosm tanks (exhibiting lengthier freeze responses; Figure 2B, left panel) and ate more (Figure 2B, right panel) than non-exposed fish. In both non-exposed and robot-exposed fish, freezing behavior and feeding rate respectively decreased and increased over time (weeks) suggesting acclimation to lab conditions, but not to the robot.

**Table 2:** Results from the fixed-factor structure of the linear-mixed models for mosquitofish, with routine activity and feeding rate as dependent variables

Model	Mean sq.	*df*	*F*	*p*
**Routine activity (s)**				

Treatment	3.551 10^2^	1,10	8.167	**0.017**
Week	1.784 10^2^	5,553	4.101	**0.001**
Trial	5.317 10^1^	1,553	1.223	0.269
Treatment × week	4.326 10^1^	5,553	0.995	0.420

**Feeding rate (counts)**				

Treatment	1.826 10^3^	1,10	1.458 10^1^	**0.003**
Week	1.360 10^3^	5,553	1.085 10^1^	**<0.001**
Trial	7.729 10^1^	1,553	0.617	0.432
Treatment × week	9.932 10^1^	5,553	0.793	0.555

Treatment (non-exposed and robot-exposed), week (two to seven), interaction (treatment × week), and trial (two repeated measures per tank per week) are included as fixed effects in each model. Random intercepts are also included for each mesocosm tank in each model, which allowed accounting for repeated measures. Analysis of variance was performed with Satterthwaite's method. Significant results are in bold.

### Life-history and fertility traits

**Figure 4:**
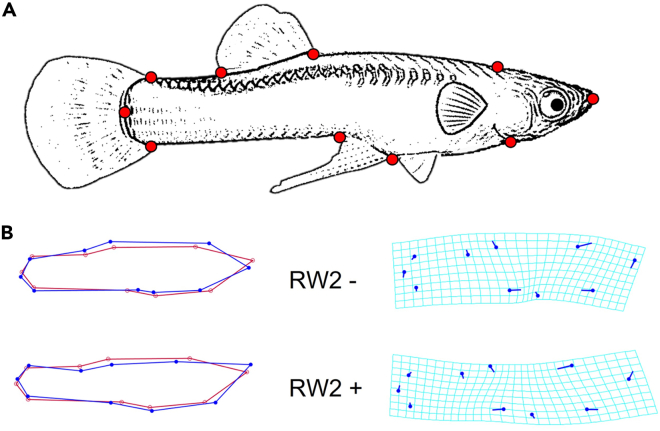
Variation in (male) mosquitofish body shape—RW2 (A) The position of ten fixed landmarks is shown on the representation of a male mosquitofish. (B) Visualization of body shape changes in RW2 from negative values (top; non-exposed fish) to positive values (bottom; robot-exposed fish) using MorphoJ software. Values are magnified 2× to help visualize differences. Red wireframe represents the consensus shape.

Non-lethal consequences of predation risk associated with the robot extended beyond behavior and altered the life-history and fertility traits of mosquitofish (Figures 2C and 4, and Tables 3 and S4). The exposure to the robot eroded energy reserves, as evident by the large reduction in their body condition (Figure 2C, left panel). A major axis of body shape changed in males (Relative Warps Score 2, RW2), but not in females, after their exposure to the robot toward a more streamlined and hydrodynamic geometry (Figure 4); other RWs were not impacted by the exposure to the robot (Table S5).

**Table 3:** Results from the fixed-factor structure of the linear-mixed models for mosquitofish, with life-history (body condition and shape) and fertility traits (sperm number and velocity in males, and egg number and weight in females) as dependent variables

Model	Mean sq.	*df*	*F*	*p*
**Body condition (*K*; g mm^−3^ 10^4^)**				

Treatment	0.019	1,57	4.070	**0.048**
Trial	0.004	1,81	0.791	0.376
Sex	0.031	1,66	6.465	**0.013**
Tank	0.007	10,57	1.502	0.162
Body weight	0.051	1,94	1.075 10^1^	**0.001**
Treatment × trial	0.001	1,67	0.268	0.607
Treatment × sex	0.002	1,57	0.347	0.558
Trial × sex	0.257	1,74	5.373 10^1^	**<0.001**
Treatment × trial × sex	0.008	1.67	1.668	0.201

**Body shape ♂ (RW2)**				

Treatment	0.001	1,23	4.527	**0.035**
Trial	0.002	1,38	1.446 10^1^	**0.003**
Tank	0.001	10,24	4.257	**0.003**
Body size	<0.001	1,36	0.006	0.593
Treatment × trial	0.001	1,34	5.197	**0.030**

**Body shape ♀ (RW2)**				

Treatment	0.001	1,57	2.973	0.126
Trial	0.004	1,57	1.858 10^1^	**0.004**
Tank	<0.001	10,57	2.259	0.055
Body size	<0.001	1,57	<0.001	0.838
Treatment × trial	<0.001	1,57	0.051	0.838

**Sperm number ♂ (counts; 10^6^)**				

Treatment	4.880 10^1^	1,12	5.653	**0.035**
Body size	0.265	1,12	0.031	0.864
Tank	0.588	1,12	0.068	0.799

**Sperm velocity ♂ (μm s^−1^)**				

Treatment	0.050	1,13	<0.001	0.990
Body size	6.924 10^1^	1,13	0.244	0.630
Tank	2.903 10^3^	1,13	1.022 10^1^	**0.007**

**Egg number ♀ (counts)**				

Treatment	1.816 10^1^	1,7	5.456	0.052
Body size	6.044 10^1^	1,7	1.816 10^1^	**0.004**
Tank	6.738	7,7	2.024	0.186

**Egg weight ♀ (mg)**				

Treatment	6.738 10^1^	1,21	7.458	**0.012**
Body size	2.190 10^2^	1,21	2.424 10^1^	**<0.001**
Tank	1.518 10^1^	10,21	1.680	0.152

Treatment (non-exposed and robot-exposed), body size/weight, and mesocosm tank are included as fixed effects in each model. Models on body shape (Relative Warps score 2, RW2) also included trial and treatment × trial as covariates, because body shape was measured twice for each individual during the study—before and after exposure to the robot—and false discovery rate-adjusted *p* values are presented. Body condition was tested on males and females together, and the model also included sex and its interaction with treatment and trial. Random intercepts (individual ID) are included in body condition and shape models, and they are absent in models for sperm and eggs as fertility traits were measured once per individual, on sexually mature individuals only. Analysis of variance was performed with Satterthwaite's method. Significant results are in bold.

Exposure to the robotic predator resulted in males exhibiting a lower sperm number compared to their non-exposed counterparts (Figure 2C, central panel), with no differences in their sperm quality (sperm velocity). Similarly, females showed a reduction in mean egg weight in response to exposure to the robot, but the variation in their egg number was marginally non-significant (Figure 2C, right panel, and Table 3).

## Discussion

We offer unequivocal evidence that is biologically inspired, interactive robotic predators can selectively control the behavior of invasive mosquitofish when cohabiting with tadpoles of native frogs. The robotic predator triggers antipredator responses in mosquitofish but not in tadpoles, and it mitigates the impact of mosquitofish on tadpoles' behavior and space use. Brief exposures to the robotic predator have long-term consequences on mosquitofish behavior—altering their routine activity and feeding rate weeks after exposure. Remarkably, these behavioral changes are accompanied by a loss of energy reserves, change in body shape, and impairment of fertility and reproductive traits in both sexes.

Robotics has emerged as a promising tool to study animal behavior and animal invasions (Krause et al., 2011; Webb, 2000), providing robots that can function autonomously (Berlinger et al., 2021; Katzschmann et al., 2018), mimic selected characteristics of live fishes (Gravish and Lauder, 2018), infiltrate social groups (Faria et al., 2010; Le Maho et al., 2014), and interact with live animals in real time (Bonnet et al., 2019; Swain et al., 2011). These robots offer a unique opportunity for biologists to study the underpinnings of behavioral responses in animals with a precise, customizable, and consistent approach that cannot be emulated with traditional methods that rely on live stimuli. The literature has documented the successful use of robotic predators to study antipredator responses in animals (reviewed in Romano et al., 2018), including individual and social responses in neon tetras (Romano and Stefanini, 2021), mosquitofish (Polverino et al., 2019; Polverino and Porfiri, 2013a; b), zebrafish (Cianca et al., 2013; Ladu et al., 2015; Nair et al., 2017; Porfiri et al., 2019), golden shiners (Swain et al., 2011), and guppies (Romano et al., 2020). Yet, the ecological and evolutionary implications of the exposure to robotic predators have remained elusive because of technical and conceptual gaps in the field of ethorobotics. Here, we fill these gaps and offer the first evidence of a robot that can selectively control the behavior of highly invasive fish, undermine the ecological success of these invaders, and alleviate their impact on native species. This evidence is likely to be particularly relevant in the context of biological invasions, because freshwater fishes and amphibians represent more than a quarter of the world's vertebrates, most of which are at risk of perishing because of the spread of invasive species (Su et al., 2021).

We developed a computer-vision system which allowed a biologically inspired robotic predator to differentiate between invasive mosquitofish and native tadpoles in real time when groups are mixed together (Figure 1). Through this system, the robot can selectively attack the invasive mosquitofish and protect native tadpoles. The robotic predator induced fear and anxiety in mosquitofish and altered their group behavior and space use, expanding evidence produced by Polverino et al. (2019) where similar acute responses were found on mosquitofish tested in isolation—a robotic predator resembling native predators in appearance, swimming speed, and acceleration, and performing controlled attacks in real time, triggers higher fear responses in mosquitofish than simpler models (Polverino et al., 2019). Our transfer entropy analysis on experimental time series indicates that the robot favored the unidirectional interaction of mosquitofish on tadpoles, meaning that behavioral adjustments allow tadpoles to escape from and not be chased by mosquitofish. These adjustments were in the form of reduced turning rates and increased spatial separation from mosquitofish, supporting the success of the robot in alleviating stress and facilitating the escape response of tadpoles. Our computer-vision system constitutes an innovation in the field of ethorobotics, offering unique opportunities for targeted experimental analyses to uncover causes and consequences of behavioral variation in animals.

Different responses of mosquitofish and tadpoles to the robotic predator can be explained by their diverse sensitivity to visual stimuli and opposing evolutionary adaptations to the predator species that inspired the design of the robot. Specifically, visual cues have a primary role for mosquitofish to avoid predators (Ward and Mehner, 2010), whereas visual acuity is weaker in tadpoles that are unable to visually detect predators unless in their close proximity (vS Hoff et al., 1999). Furthermore, the appearance and motion of the robotic predator are inspired by largemouth bass (*Micropterus salmoides*), the mosquitofish's primary natural predator in its native environment (García-Berthou, 2002; Wicker and Johnson, 1987), but unknown to Australian tadpoles. Because tadpoles do not share an evolutionary history with such a predator, it is plausible that they do not recognize it as a threat (Hossie et al., 2017). Overall, we provide new evidence that state-of-the-art robots can selectively control target species in the ecological community in which they inhabit.

Certain life experiences are crucial for the behavioral development of an individual, and can shape behavioral strategies that persist over time (Panchanathan and Frankenhuis, 2016). For instance, failing to obtain a meal or mating can have minimal or no influence on an individual's lifetime fitness, but failing to survive a predatory attack decreases future fitness substantially. Therefore, predation risk requires animals to adjust their future behavior, independent of actual predation (Lima, 1998a). Yet, predatory behaviors in live predators vary extensively, depending on their motivation (e.g., hunger levels) and the risk-taking behavior of the prey. Our predator-mimicking robot enabled us to overcome these challenges through targeted vision- and motion-based predatory cues, and to precisely quantify the consequences of non-lethal exposures on fish behavior weeks after exposure. Our results revealed that behavioral changes of mosquitofish in the experimental arena while in the presence of the robot carried over to influence their routine behavior in the mesocosm tanks*—*brief and non-lethal exposures to the robotic predator translate into increased antipredator behaviors and feeding rates in mosquitofish in the long term. The risk of an early death is indeed expected to favor cautious behaviors (low activity levels) and maximize energy intake when predators are absent (Lima, 1998b), moderating the trade-off between reducing predation risks and fueling growth and reproductive fitness. Classic mathematical models support this empirical evidence and align with our finding, indicating that costs of antipredatory efforts can be adaptively thwarted if animals increase their feeding rate when resources are available for brief periods at low predation risk (Lima and Bednekoff, 1999).

Interestingly, the antipredator responses triggered by the robot had negative repercussions on mosquitofish life-history (energy reserves) and reproductive traits (sperm and eggs), and such long-term costs of perceived predation risk were not fully counterbalanced by increased feeding rates. This suggests that mosquitofish exposed to the robotic predator allocated relatively more energy to antipredator functions, at the costs of energy reserves and reproduction. According to theory, a trade-off between survival and future reproduction is, indeed, predicted for animals under high risk of predation (Lima, 1998a; b). Previous research has reported that visual exposure to live largemouth bass*—*the main native predator of mosquitofish, which has inspired both the appearance and motion of our robotic predator*—*is responsible for lower body condition and fertility in female mosquitofish (Mukherjee et al., 2014). These prior findings support the idea that mosquitofish perceived the robot as a real predator. To what extent and for how long the observed reduction in sperm number (males) and egg weight (females) compromised reproduction in mosquitofish remains to be tested directly. However, previous studies have determined that lower sperm number predicts lower fertilization success in male mosquitofish (Head et al., 2015), and that risk-induced responses in females cause smaller clutch size and alter life histories of their offspring (Mukherjee et al., 2014).

Taken together, our results indicate that a relatively brief exposure to a robotic predator has consequences for mosquitofish phenotypes spanning from behavior to life history and reproduction. The long-term impact of the robotic predator on mosquitofish life-history strategy is further supported by the alteration of their body shape, which occurred in only five weeks. Males exposed to the robot developed more streamlined and hydrodynamic body shapes, which are known to enhance locomotor performance and escape abilities in fishes, and are typical of mosquitofish living under high predation risk in the wild (Langerhans et al., 2004). Variation between treatments in females' body shape was not detected, most likely because all females developed larger abdomens during the experiment (egg production and pregnancy; Belk and Tuckfield, 2010), thus potentially masking the effects of predation risk on changes in their shape. Therefore, exposure to biologically inspired, interactive robots has the potential to reshape the life-history strategies of animals, impacting their survival and reproduction in the long term.

The interactive nature of the robotic predator reduces the risk of fish habituating to the setup by randomizing the display of different attacks, a key advantage from the use of real-time interactions with live animals (Krause et al., 2011). Although on average fish decreased their stress-related behaviors and increased feeding rate throughout the study, such changes did not differ between non-exposed and robot-exposed mosquitofish. This evidence indicates that our wild-caught fish acclimated to lab conditions but not to the interactive robotic predator*—*at least within the time frame considered in this study.

In conclusion, our study reveals the costs of non-lethal stress—in the form of finely controllable and biologically inspired robotic predators—in terms of behavior, life history, reproductive traits, and ecological success of a damaging invasive species. Reducing the impact of invasive species is a priority of both governmental and non-governmental institutions worldwide, but it remains a significant challenge that demands fresh approaches. Understanding the ecological and evolutionary factors underlying the success of invasive species is a crucial first step toward developing effective biosecurity and management strategies.

### Limitations of the study

We focused on mosquitofish as a target species because it is a model system in ecology and evolution, and is a serious threat to biodiversity globally. But predation is a problem for all species, and fear has general implications across animals, not just mosquitofish or fishes in general. Hence, the fundamental knowledge on predator–prey ecology generated in this work should apply far beyond mosquitofish, but further analyses are needed for this to be verified.

Our technology, at present, does not offer a solution to eradicate mosquitofish in the wild, and its effective deployment in natural settings will entail substantial technical and conceptual challenges.

In spite of these limitations, we expect our work to set the stage for research on other target species and future technological applications.

## STAR★Methods

### Key resources table

**Table:** 

REAGENT or RESOURCE	SOURCE	IDENTIFIER	
**Biological samples**			

*Gambusia holbrooki* sperm	This paper	N/A	
*Gambusia holbrooki* eggs	This paper	N/A	

**Critical commercial assays**			

Robotic platform (XY Plotter Robot Kit)	Makeblock Co., Ltd, Shenzhen (China)	V2.0	
Microcontroller (Arduino Leonardo)	Interaction Design Institute Ivrea (Italy)	https://www.arduino.cc/en/Main/Arduino_BoardLeonardo	
Computer-assisted sperm analyses (CEROS sperm tracker)	Hamilton-Thorne Research (USA)	www.hamiltonthorne.com	

**Deposited data**			

Raw data	This paper	https://doi.org/10.6084/m9.figshare.14420639	
Codes for statistical analyses	This paper	https://doi.org/10.6084/m9.figshare.14420639	

**Experimental models: Organisms/strains**			

Adult/subadult fish (*Gambusia holbrooki*)	Herdsman Lake and Jualbup Park (Australia)	N/A	
Frog tadpoles (*Litoria moorei*)	Ephemeral ponds (Australia)	N/A	

**Software and algorithms**			

Real-time tracking software	This paper		N/A
GRBL 0.9 software	Simen Svale Skogsrud (http://bengler.no/grbl)		https://github.com/grbl/grbl
Solidworks software (3D CAD Design)	Dassault Systèmes SolidWorks Corp., Waltham (USA)		https://www.solidworks.com/
Munkres algorithm	Journal of the Society for Industrial and Applied Mathematics		https://doi.org/10.1137/0105003
tpsDig2 software (landmarks for shape analysis)	State University of New York at Stony Brook (USA)		https://sbmorphometrics.org/
tpsRelW software (relative warps for shape analyses)	State University of New York at Stony Brook (USA)		https://sbmorphometrics.org/
MorphoJ software (shape analyses)	Molecular Ecology Resources		https://doi.org/10.1111/j.1755-0998.2010.02924.x
lmerTest (R package)	https://cran.r-project.org/		https://www.jstatsoft.org/article/view/v082i13
nlme (R package)	https://cran.r-project.org/		http://CRAN.R-project.org/package=nlme
emmeans (R package)	https://cran.r-project.org/		https://cran.r-project.org/web/packages/emmeans/index.html

### Resource availability

#### Lead contact

Further information and requests for resources, data, and codes should be directed to and will be fulfilled by the lead contact, Giovanni Polverino (gio.polverino@gmail.com).

#### Materials availability

This study did not generate new unique reagents.

### Experimental model and subject details

The mosquitofish (*Gambusia holbrooki*) provides an ideal model system for studying behaviour, ecology, and evolution, and was chosen for this study based on the following grounds: biologically inspired robotic predators have been shown to affect mosquitofish behaviour (Polverino et al., 2019), the species is amenable to both captive and field-based studies (Pyke, 2008), and the link between behaviour, survival, and reproduction in this species has been established (Polverino et al., 2018).

We collected 150 mosquitofish from two permanent lakes in Western Australia, Herdsman Lake and Jualbup Park, and 100 tadpoles of the native motorbike frog (*Litoria moorei*) from ephemeral and mosquitofish-free ponds adjacent to these locations. Animals were collected and assayed during the Australian winter (June to August), prior to their breeding season. We housed mosquitofish and tadpoles in separate stock tanks with gravel substrates, aeration and filtration systems, large rocks, and live vegetation from their native environment. After 24h of acclimation, 72 young-adult mosquitofish and 72 tadpoles at similar age (mean body size in mm ± SE for fish: 21.32 ± 0.28, tadpoles: 59.00 ± 1.30) were selected haphazardly from stock tanks and distributed across 12 identical mesocosm tanks randomly.

Six mosquitofish (three females and three males) and six tadpoles were housed in each mesocosm tank (44 × 42 × 33 cm, length, width, and height), filled with 27 cm of conditioned water. One third of the water in each mesocosm tank was changed every week to maintain high water quality. Each mesocosm tank was equipped with aeration and filtration systems, large rocks, and live vegetation to resemble the native habitat of both species. We covered the sides of each mesocosm tank for visual isolation and to minimise external disturbance. All mesocosm tanks were housed in a temperature-controlled room (22.5 ± 1⁰ C) with a 12:12 h light:dark cycle throughout the study.

At the start of the experiment, at least one fully mature male mosquitofish was placed in each mesocosm tank, and males at the onset of sexual maturity (subadults) were distributed randomly.

Animals were fed with commercial flake food (Nutrafin max; Hagen Corp., Mansfield, MA, USA) six days per week. Mosquitofish also received live *Artemia salina* nauplii four days per week during feeding trials (details below), while tadpoles were supplemented with boiled lettuce daily.

Experiments were performed in accordance with relevant (ARRIVE) guidelines and recommendations, and were approved by the Animal Ethics Office of the University of Western Australia under the permit number RA/3/100/1663, and by the University Animal Welfare Committee of the New York University under 13-1424.

### Method details

#### Robotic predator and group-behaviour measures

##### Setup

We assayed behaviour in mixed groups of mosquitofish and tadpoles—each mesocosm tank separately—in a white experimental arena (42 × 35 cm, diameter × height) filled to a depth of 10 cm with conditioned water (Figure 1), as per established protocols (Polverino et al., 2018, 2019). We mounted two high-resolution webcams 135 cm above the arena for a complete view, and three 60W lamps for homogenous illumination.

The arena was positioned on a supporting structure 30 cm above the ground, and our previously developed robotic manipulator (Polverino et al., 2019) was placed underneath (Figure 1). The robotic platform integrates real-time tracking and high-precision robotics to allow for finely controlled movements of the robotic predator, comprising in-plane manoeuvers and body rotations. For behavioural assays on treatment (robot-exposed) groups, the robotic predator was placed inside the arena and connected to the platform with two magnets and a clear acrylic rod (Figure 1). Mixed groups of mosquitofish and tadpoles from the robot-exposed treatment were exposed to the robotic predator, while control (non-exposed) groups were tested in the area without the robotic predator.

Our robotic platform comprised three components: a high-precision Cartesian manipulator, a biologically inspired robotic predator, and a real-time tracking system. The robotic manipulator was a commercial Cartesian plotter (XY Plotter Robot Kit, Makeblock Co., Ltd, Shenzhen, China), fitted with three stepper motors (NEMA 14, Pololu Corp., Las Vegas, NV, USA) to control translation along the X-Y Cartesian plane and to control body rotation of the robotic predator along its axis. We used a microcontroller (Arduino, Ivrea, Italy) and a motor shield (Kuman CNC Kit, Kuman Trade Co., Shenzhen, China) to drive the stepper motors with sub-millimeter accuracy and interface the manipulator with the computer. We utilised the GRBL 0.9 software library (Sungeun and Skogsrud, 2011) and MATLAB R2019a (The MathWorks, Inc., Natick, MA, USA) to establish a serial communication between the computer and the robotic platform, sending data consisting of position, speed, and turning rate of the animals to the platform to inform the real-time interaction with the robotic predator.

The appearance and motion patterns of the robotic predator were inspired by the primary predator that coevolved with mosquitofish in its native environment—juvenile largemouth bass—building on prior knowledge and experimental validation (Polverino et al., 2019). Briefly, the body of the robotic predator was made of non-toxic silicon, moulded to a 3D printed polylactic filament spine-like structure, and dyed with non-toxic paints to mimic the characteristic coloration pattern of the largemouth bass. The motion of the robotic predator was inspired by trajectories, speed, and acceleration of live bass, including those associated with attacks towards mosquitofish (Polverino et al., 2019). Previous evidence confirmed that antipredator responses in mosquitofish increase with increasing biomimicry of the robotic predator, including: body morphology (Polverino and Porfiri, 2013a), colouration (Polverino and Porfiri, 2013b), and swimming patterns (Polverino et al., 2019). The swimming depth was set to be in the middle of the water column, which is also known to cause stronger antipredator responses in mosquitofish (Polverino and Porfiri, 2013a).

We developed a multi-species, live-tracking system using the computer vision toolbox in MATLAB R2019a (The MathWorks, Inc., Natick, MA, USA), which allowed the robot to discriminate between mosquitofish and tadpoles in real time, and interact with each species differently (Figure 1). The tracking system integrated a body characteristic-based filtering algorithm with a multiple thresholding strategy to precisely distinguish between mosquitofish and tadpoles according to their coloration, size, and movement pattern. The system automatically identified the species of each individual and stored its centroid location in real time. Thresholding strategies were implemented on three initial frames to optimise the detection of each species while also preventing interference from the motion of the robotic predator. After filtering noise, blob analyses were performed on each frame—in which connected pixels were identified, and their centroids tracked independently for each species. These centroids were then related to each individual using the Munkres' assignment algorithm (Munkres, 1957), with individuals of both species tracked simultaneously. When an individual was not detected, its position was estimated through a Kalman predictor associated with that individual, which was informed by the history of its trajectory under constant velocity assumption (Blackman, 1986). During each group-behaviour assay (60 min), the tracking system ran in real time at 20 frames per second.

For the five weeks in which mosquitofish and tadpoles were exposed to the robotic predator (robot-exposed treatment), the robot attacked the mosquitofish that was closest to the tadpoles at a frequency of approximately one attack per minute. Attacks varied in length depending on the distance between the robot and the targeted mosquitofish. So the total number of attacks was fewer than 60, and was standardized across trials to either be 55 (26 trials) or 56 (34 trials) attacks per trial. If the targeted mosquitofish was within 1 cm from the robot, the robotic predator changed heading toward the fish and waited for 1 s before returning to its initial heading. If the targeted mosquitofish was between 1 and 10 cm (inspection zone; Magurran and Seghers, 1990), the robotic predator accelerated at 20 cm/s^2^ and stopped at approximately 1 cm, waited for 1 s, and then returned to its initial position and heading. If the targeted mosquitofish was further than 10 cm, the robotic predator accelerated at 20 cm/s^2^ until reaching its maximum speed of 20 cm/s, stopped 1 cm away from the fish, waited for 1 s, and then returned to its initial position and heading. After an attack was completed, the robotic predator returned to its initial position and resumed swimming along the predetermined trajectory—inspired by swimming trajectories observed in live largemouth bass (Polverino et al., 2019)—until the next attack.

##### Procedure

From week one to week seven, each mixed group of mosquitofish and tadpoles was behaviourally assayed in the experimental arena twice a week (three/four days apart), for a total of 14 trials per group. At each given trial, we gently hand-netted the group of mosquitofish and tadpoles from their mesocosm tank and transferred them into the experimental arena. After 1 min of acclimation, a trial was initiated and lasted for 60 min. During acclimation, the motors of the robotic platform were turned off and animals had no visual contact with the apparatus or the robotic predator. For the treatment (robot-exposed) groups, we introduced the robotic predator in the arena and turned on the platform soon after the acclimation ended—except for week one and seven, in which all groups were tested in the absence of the robotic predator. In contrast, the group behaviour of mosquitofish and tadpoles from the non-exposed treatment was measured in the absence of the robotic predator. After a trial was completed, the animals were transferred back into their mesocosm tank, and the experimental arena was drained, washed, and refilled with conditioned water to prevent chemical interactions between groups. The order of trials was randomised every day to prevent biases due to hunger (Krause et al., 1998).

##### Group-behavioural assays

Our multi-species, live-tracking system supplied the information for the robot to interact with live animals in real time (Figure 1). At the same time, the tracking system recorded the individual behaviour of all mosquitofish and tadpoles in the mixed group simultaneously (*n* = 12), and allowed us to explore the group dynamics for each species separately, as well as their interactions.

We focused on the average furthest neighbour distance (AFND; average distance between an individual and its furthest neighbour, in cm) and average inter-individual distance (AIID; average distance between all possible pairs of individuals within the group, in cm) to measure group cohesion and estimate social interactions within and between species.

We quantified the overall activity of each species in terms of the average distance swam (in cm) and average turning rate (in rad s^−1^) in each trial separately. By acquiring both these kinematic measurements, we can disentangle instances in which animals swam long trajectories by erratically moving in a small portion of the tank (large average distance and large average turning rate) versus instances in which they explored the entire tank without displaying sudden, frequent turning manoeuvres (large average distance and small average turning rate).

We also measured the average time spent in the central and external regions of the arena (in s) for each species separately at each given trial, thereby capturing variation in their space use. We initially partitioned the arena into seven concentric regions increasing in diameter by 3 cm, and we analysed spatial preferences for each species across all regions. However, our data clearly showed that variation in the space use of both mosquitofish and tadpoles was better explained by condensing the seven regions into two: the central (0–15 cm radius) and the external region (15–21 cm radius). Further details are in the supplemental information (Figure S1).

#### Feeding rate and routine activity

Beyond the acute effect of predator exposure on group behaviour, we tested whether effects carried over onto feeding rates and routine activity of mosquitofish in the mesocosm tanks after the exposure—low feeding rates and routine activity are often associated with fearful and anxiety-related states in fishes (Maximino et al., 2010; Schröder et al., 2016). We performed feeding and routine activity assays on each mosquitofish group twice a day for four days a week (week one to seven), for a total of 112 trials on each independent group for both behaviours.

A standard amount of live *Artemia salina* nauplii was offered in the morning to mosquitofish in each mesocosm tank. Soon after, their feeding rate was measured as the number of nauplii eaten by a randomly chosen mosquitofish within a one-minute period. For each mesocosm tank, we also measured routine activity as the amount of time that a randomly chosen mosquitofish was inactive (“freezing”, in s) within a five-minute period; freezing is defined as immobility for more than one second, and it is commonly used to quantify stress in fishes. We randomised the order of mesocosm tanks assayed for feeding rates and routine activity every day.

We allowed a minimum of one hour between the feeding trials and routine activity assays, and one and a half hours between routine activity trials and group-behaviour assays in the experimental arena.

#### Life-history and fertility traits

Before the study began and after its conclusion, mosquitofish were anaesthetised in a solution of AQUI-S (20 mg L^−1^), photographed, sexed, and measured for their standard body size (to the nearest 0.01 mm) and weight (to the nearest 0.01 g)—two measurements per individual in total. We calculated the Fulton's condition factor *K* (weight length^−3^ 10^4^, g mm^−3^ 10^4^; Froese, 2006) of each individual as an index for its body condition. Variation in the body shape of each individual mosquitofish was analysed using geometric morphometric landmark-based methods (males *n* = 35; females *n* = 36), as described previously for this species (Langerhans et al., 2004). This process consisted of two steps: first, we digitised 10 landmarks at homologous (fixed) points on each image via the tpsDig2 software (Rohlf, 2005a), and then we used relative warps analyses (tpsRelW; Rohlf, 2005b) to reduce multivariate shape data to relative warps (RWs) that describe the majority of variation in body shape across our sample. Males and females were analysed separately. Four RW scores for females and three RW scores for males, explaining >70% of variation in shape in both sexes, were retained for our analyses of shape (Table S6). Shape variation was visualised using the software MorphoJ (Klingenberg, 2011; Figure 4).

At the end of the study, we assessed the fertility traits of sexually mature male and female mosquitofish from both the robot-exposed (males *n* = 8; females *n* = 17) and non-exposed treatment (males *n* = 8; females *n* = 17). Sperm number and sperm velocity were assessed for each male using methods specifically developed for poeciliid fishes (Gasparini et al., 2017). Briefly, each male was first anaesthetised, and sperm were collected after applying gentle pressure to the abdomen. Sperm were counted with a Neubauer haemocytometer, and sperm velocity was assessed using a Hamilton-Thorne CASA system (sperm tracker). Sperm velocity analyses were based on an average of 223 motile sperm per sample. All males were then sacrificed with an excess of AQUI-S anaesthetic. Females were sacrificed at first, and their body cavities dissected to assess the number and dry weight of eggs, following established protocols (Polverino et al., 2018).

### Quantification and statistical analysis

Data analyses were performed in *R* using the packages *lmerTest*, *nlme*, and *emmeans* (Kuznetsova et al., 2013; Lenth, 2018; Pinheiro et al., 2014).

We tested whether the robotic predator altered the group-behaviour dynamics between mosquitofish and tadpoles when mixed together, specifically the extent to which the robot altered sociality, activity, and space use in both species. Accordingly, we analysed sociality by fitting univariate linear mixed-effects models (LMMs) for AFND and AIID for mosquitofish, tadpoles, and mixed groups of mosquitofish and tadpoles separately. We tested activity by fitting LMMs with distance swam and turning rate for mosquitofish and tadpoles separately. Finally, we tested space use in mosquitofish and tadpoles separately by including time spent in the central region of the arena—which mirrors the time spent in external region—as the dependent variable in a LMM. Fixed effects included in each model were treatment (robot-exposed and non-exposed), week (two to six), interaction (treatment × week), and trial (two repeated measures per tank per week), while mesocosm tank identities (*n* = 12; six per treatment) were included in the random structure (random intercepts) to account for repeated measures. Six replicates per treatment are sufficient to obtain statistical power and test variation in phenotypic traits in mosquitofish (Cote et al., 2010). Through the interaction effect between treatment and week, we tested for the possibility that group-behaviour dynamics differed between treatments over time, that is, habituation to the setup differed between robot-exposed and non-exposed groups. Week one referred to assays performed before robot exposures started, and it was tested separately, revealing that no differences existed between mesocosm tanks before treatments commenced (Table S7).

Following established practice (Porfiri, 2018), we computed transfer entropy (Schreiber, 2000) between the time series of the group turning rate of the mosquitofish and tadpoles (Figures 3A and 3C). Specifically, each time series consisted of a sequence of binary symbols, indicating whether the average magnitude of the turning rate in the group increased or not between two consecutive instants half a second apart in time. In addition to the study of interactions between mosquitofish and tadpoles with respect to swimming patterns, we pursued an equivalent transfer entropy analysis to ascertain interactions in terms of space use. Transfer entropy was computed on time series of binary symbols describing whether the number of animals (mosquitofish or tadpoles) in the central region of the experimental arena increased or not between acquired time steps half a second apart (Figures 3B and 3D). For each trial, we used a time series of over 7,000 points per individual. Each raw time series was converted into a time series of binary symbols, where symbol “+” encoded an increase in between two consecutive time steps and symbol “–” captured instances in which there was either no change or there was a decrease in between two consecutive time steps. Specifically, we computed transfer entropy from mosquitofish (M) to tadpoles (T) as follows:

$${\text{TE}}_{\text{Mosq}\to \text{Tadp}}=\sum \text{T}\left(\text{t}+1\right)\text{,T}\left(\text{t}\right)\text{,M}\left(\text{t}\right)\text{Pr}\left(\text{T}\left(\text{t}+1\right)\text{,T}\left(\text{t}\right)\text{,M}\left(\text{t}\right)\right){\log\;}_{2}\frac{\mathrm{Pr}\left(\text{T}\left(\text{t}+1\right)|\text{T}\left(\text{t}\right)\text{,M}\left(\text{t}\right)\right)}{\mathrm{Pr}\left(\text{T}\left(\text{t}+1\right)|\text{T}\left(\text{t}\right)\right)},$$

where T and M are symbolic time series of the average magnitude of the turning rate or the number of animals in the central region, t is the time step, and Pr depicts the probability mass function calculated by plug-in estimation. Transfer entropy from the tadpoles to the mosquitofish ${\text{TE}}_{\text{Tadp}\to \text{Mosq}}$ was analogously computed by inverting T with M. We calculated transfer entropy between the two species for both treatments (non-exposed and robot-exposed) and across five weeks of behavioural trials in the experimental arena (week two to six). Statistical significance of our results was assessed via permutation tests, where we randomly shuffled the identities of the groups from different trials 20,000 times to create a null distribution. We then ascertained whether the experimental mean transfer entropy value was within the 95% quantile of the null distribution to pinpoint significant interactions.

Our primary goal was to test whether brief exposures to the robotic predator had long-term effects on the routine behaviour, life history, and reproductive traits of mosquitofish. For the routine behaviour, we tested LMMs separately with routine activity (freezing) and feeding rate as the dependent variables, and included fixed and random effects in each model as described above: treatment, week, interaction, and trial were included as fixed effects, while mesocosm tank identities were included as random intercepts.

To test whether fish from different treatments differed in their life history and fertility, we fitted LMMs separately for each of the following traits: body size, weight, condition, and shape, sperm number and velocity (males), and egg number and weight (females). LMMs for body size and weight included individual identity as the random effect (random intercepts), and treatment, trial, sex, treatment × trial × sex interaction, and mesocosm tank as fixed effects. The LMMs for body condition and shape also included body size as an extra covariate to account for variation in size between individuals; only the first four RWs for females and three RWs for males were tested (one-by-one), since together they explained >70% of variation in shape in both sexes (Table S6), and multiple comparisons were corrected with the false discovery rate procedure to control for type I and type II errors (Pike, 2011). Since our fertility estimates relied on one data point per individual, LMMs for fertility included treatment, tank, and body size as fixed effects, and mesocosm tank identities as random intercepts.

We assumed Gaussian error distribution, which was confirmed for all response variables after visual inspection of model residuals. We ran pairwise comparisons with the conservative Bonferroni method for significant predictors in each LMM and accounted for the variation explained by other predictors. The significance level was set at *α* < 0.05.

## Data Availability

• All data have been deposited at Figshare repository, and are publicly available as of the date of publication at Database: https://figshare.com/articles/dataset/Ecology_of_fear_in_highly_invasive_fish_revealed_by_robots/14420639. Accession numbers are also listed in the key resources table. • All original codes have been deposited at Figshare repository, and are publicly available as of the date of publication at Codes: https://figshare.com/articles/dataset/Ecology_of_fear_in_highly_invasive_fish_revealed_by_robots/14420639. DOIs are listed in the key resources table. • Any additional information required to reanalyse the data reported in this paper is available from the lead contact upon request.
