# Supplementary material for: Ecology of fear in highly invasive fish revealed by robots
Source: iScience. 2021 Dec 16;25(1):103529. doi: 10.1016/j.isci.2021.103529 (PMC8786638; doi:10.1016/j.isci.2021.103529)
Supplement: Document S1. Figure S1 and Tables S1–S7 [file mmc1.pdf]

iScience, Volume *25*

Supplemental information

Ecology of fear in highly invasive fish revealed by robots

Giovanni Polverino, Vrishin R. Soman, Mert Karakaya, Clelia Gasparini, Jonathan P. Evans, and Maurizio Porfiri

This PDF file includes

Fig. S1. Average space use of mosquitofish and tadpoles in the experimental arena, Related to Fig. 2a, and Table 1.
Table S1. Results from the random-factor structure of the linear-mixed models on group-behaviour measures, Related to Fig. 2a and Table 1.
Table S2. Transfer entropy between mosquitofish and tadpoles within each treatment, Related to Fig. 3.
Table S3. Results from the random-factor structure of the linear-mixed models for mosquitofish, with routine activity and feeding rate as dependent variables, Related to Fig. 2b, and Table 2.
Table S4. Results from the random-factor structure of the linear-mixed models for mosquitofish, with body condition and shape (Relative Warps score 2, RW2) as dependent variables, Related to Fig. 2c and 4, and Table 3.
Table S5. Results from linear-mixed models for mosquitofish, with body shape (Relative Warps score, RWs) tested separately for males (RW1 and RW3) and females (RW1, RW3, and RW4) as dependent variables, Related to Fig. 4.
Table S6. Relative Warps scores (RWs) describing variation in body shape of male and female mosquitofish separately, Related to Fig. 4.
Table S7. Results from univariate linear-mixed models on group-behaviour measures before treatments started (i.e. week one), Related to Fig. 2a and Table 1.

FIGURES

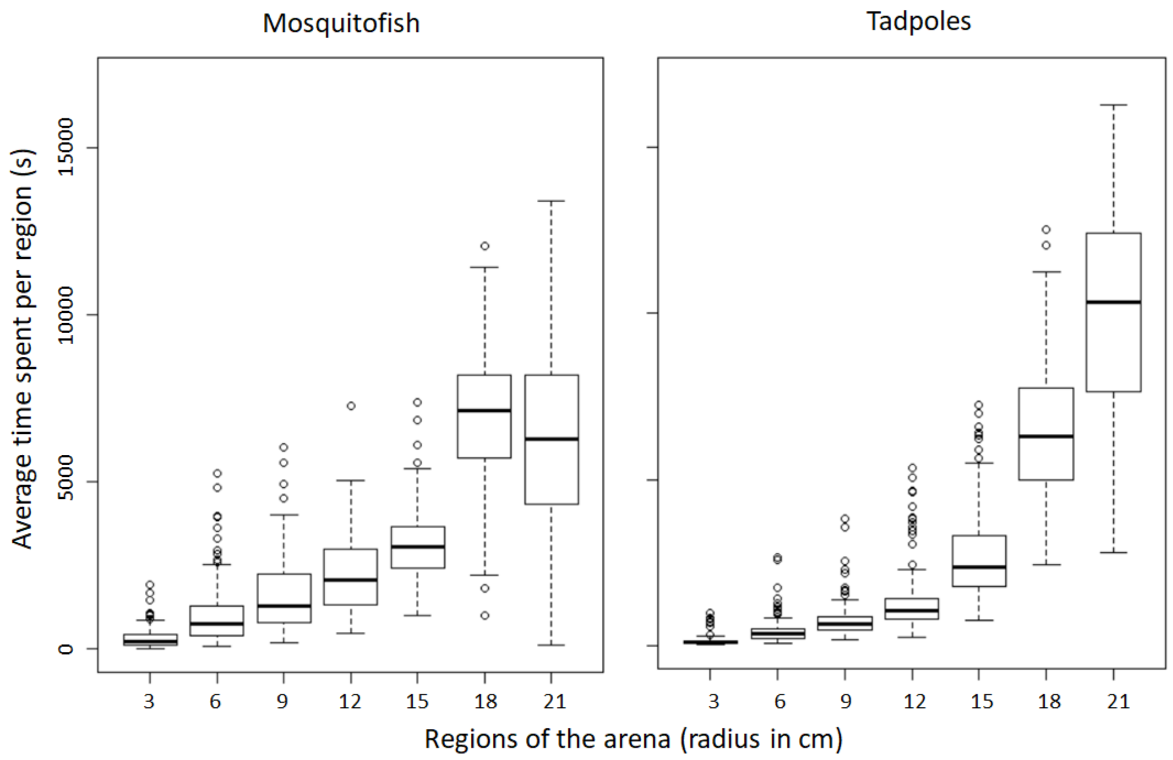

Fig. S1. Average space use of mosquitofish and tadpoles in the experimental arena, Related to Fig. 2a, and Table 1. The space use was calculated separately for each species by dividing the circular arena in seven concentric zones, each one of 3 cm in radius. These preliminary analyses indicated comparable scores for the time spent in regions between 0-15 and 15-21 radiuses from the centre of the arena for both species. Thus, intermediate regions were condensed into two main regions: a central (0-15 cm radius) and an external region (15-21 cm radius).

TABLES

Table S1. Results from the random-factor structure of the linear-mixed models on group-behaviour measures, Related to Fig. 2a and Table 1. The dependent variables, cohesion (average furthest neighbour distance, AFND, and average inter-individual distance, AIID), activity (turning rate and distance swam), and space use (time in the central region), are tested separately. Treatment (non-exposed and robot-exposed), week (two to six), interaction, and trial (two repeated measures per tank per week), are included as fixed effects in each model. Random intercepts are also included in each model to account for repeated measures of mesocosm tanks.

	Mosquitofish		Tadpoles		Mosquitofish & tadpoles	
Model	Variance	SD	Variance	SD	Variance	SD
AFND						
Tank	0.217	0.465	0.046	0.215	0.331	0.575
Residuals	5.350	2.313	2.078	1.441	2.630	1.622
AIID						
Tank	0.130	0.361	0.061	0.248	0.212	0.461
Residuals	2.472	1.572	1.044	1.022	1.417	1.190
Turning rate						
Tank	0.006	0.077	0.015	0.125	-	-
Residuals	0.042	0.206	0.246	0.496	-	-
Distance Swam						
Tank	$1.814\ 10^{7}$	$4.259\ 10^{3}$	$1.500\ 10^{7}$	$3.873\ 10^{3}$	-	-
Residuals	$4.789\ 10^{8}$	$2.188\ 10^{4}$	$2.428\ 10^{8}$	$1.558\ 10^{4}$	-	-
Time in central region						
Tank	$1.985\ 10^{6}$	$1.409\ 10^{3}$	0.001	0.001	-	-
Residuals	$7.030\ 10^{6}$	$2.651\ 10^{3}$	$4.382\ 10^{6}$	$2.093\ 10^{3}$	-	-

Table S2. Transfer entropy between mosquitofish and tadpoles within each treatment, Related to Fig. 3. Computation use binary symbolic time series of average magnitude of group turning rate and presence in the central region of the arena. Values, in bits, indicate the mean transfer entropy across trials for each treatment separately, with 95% percentile of the surrogate data in parentheses. Significant results are in bold.

	Non-exposed		Robot-exposed	
	$TE_{Mosq \rightarrow Tadp}$	$TE_{Tadp \rightarrow Mosq}$	$TE_{Mosq \rightarrow Tadp}$	$TE_{Tadp \rightarrow Mosq}$
Turning rate	$2.24\ 10^{-4}$<br>($2.57\ 10^{-4}$)<br>$p = 0.303$	$1.73\ 10^{-4}$<br>($2.59\ 10^{-4}$)<br>$p = 0.930$	$2.53\ 10^{-4}$<br>($2.52\ 10^{-4}$)<br>$p =$ **0.040**	$1.90\ 10^{-4}$<br>($2.45\ 10^{-4}$)<br>$p = 0.633$
Presence in central region	$1.66\ 10^{-3}$<br>($1.58\ 10^{-3}$)<br>$p <$ **0.001**	$1.81\ 10^{-3}$<br>($1.75\ 10^{-3}$)<br>$p <$ **0.001**	$1.58\ 10^{-3}$<br>($1.57\ 10^{-3}$)<br>$p =$ **0.024**	$1.48\ 10^{-3}$<br>($1.54\ 10^{-3}$)<br>$p = 0.623$

Table S3. Results from the random-factor structure of the linear-mixed models for mosquitofish, with routine activity and feeding rate as dependent variables, Related to Fig. 2b, and Table 2. Treatment (non-exposed and robot-exposed), week (two to seven), interaction, and trial (two repeated measures per tank per week) are included as fixed effects in each model. Random intercepts are also included in each model to account for repeated measures of mesocosm tanks.

Model	Variance	SD
Routine activity		
Tank	2.197	1.482
Residuals	4.348 10^{1}	6.594
Feeding rate		
Tank	3.671	1.916
Residuals	1.253 10^{2}	1.119 10^{1}

Table S4. Results from the random-factor structure of the linear-mixed models for mosquitofish, with body condition and shape (Relative Warps score 2, RW2) as dependent variables, Related to Fig. 2c and 4, and Table 3. Treatment (non-exposed and robot-exposed), body size/weight, mesocosm tank, trial (two repeated measures per tank per week), and treatment × trial are included as fixed effects in each model. Body condition was tested for males and females together, so models also included sex and its interaction with treatment and trial. Random intercepts are also included in each model to account for repeated measures of individual IDs.

Model	Variance	SD
Body condition		
Individual	0.009	0.097
Residuals	0.005	0.069
Body shape ♂ RW2		
Individual	$2.172\ 10^{-5}$	0.005
Residuals	$1.568\ 10^{-4}$	0.012
Body shape ♀ RW2		
Individual	<0.001	<0.001
Residuals	<0.001	0.015

Table S5. Results from linear-mixed models for mosquitofish, with body shape (Relative Warps score, RWs) tested separately for males (RW1 and RW3) and females (RW1, RW3, and RW4) as dependent variables, Related to Fig. 4. Treatment (non-exposed and robot-exposed), trial (two repeated measures for each individual—before and after treatment), treatment × trial, body size, and mesocosm tank are included as fixed effects, and random intercepts (individual ID) are also included in each model. Analysis of variance was performed with Satterthwaite's method, and false discovery rate adjusted p-values (*p*) are presented.

Model				
RW1 ♂	Mean sq.	*df*	*F*	*p*
Treatment	<0.001	1,22	0.125	0.763
Trial	<0.001	1,37	0.694	0.717
Tank	<0.001	10,23	0.777	0.763
Body size	0.001	1,53	5.184	0.142
Treatment × trial	<0.001	1,32	1.363	0.661
	Variance	SD		
Individual	<0.001	0.021		
Residuals	<0.001	0.017		
RW1 ♀	Mean sq.	*df*	*F*	*p*
Treatment	<0.001	1,23	1.555	0.394
Trial	<0.001	1,37	2.109	0.394
Tank	<0.001	10,23	0.437	0.959
Body size	0.001	1,28	3.715	0.336
Treatment × trial	<0.001	1,34	0.170	0.896
	Variance	SD		
Individual	<0.001	0.012		
Residuals	<0.001	0.017		
RW3 ♂	Mean sq.	*df*	*F*	*p*
Treatment	<0.001	1,22	0.043	0.880
Trial	<0.001	1,37	3.997	0.278
Tank	<0.001	10,23	1.521	0.458
Body size	<0.001	1,34	1.302	0.458
Treatment × trial	<0.001	1,33	0.335	0.744
	Variance	SD		
Individual	<0.001	0.004		
Residuals	<0.001	0.012		
RW3 ♀	Mean sq.	*df*	*F*	*p*
Treatment	<0.001	1,23	3.346	0.210
Trial	0.001	1,36	5.942	0.105
Tank	<0.001	10,23	0.879	0.973
Body size	<0.001	1,27	0.003	0.999
Treatment × trial	<0.001	1,34	0.111	0.973
	Variance	SD		
Individual	<0.001	0.006		
Residuals	<0.001	0.014		
RW4 ♀	Mean sq.	*df*	*F*	*p*
Treatment	<0.001	1,57	1.153	0.398
Trial	<0.001	1,57	4.625	0.094
Tank	<0.001	10,57	0.275	0.999
Body size	0.001	1,57	6.539	0.068
Treatment × trial	<0.001	1,57	1.078	0.398
	Variance	SD		
Individual	<0.001	<0.001		
Residuals	<0.001	0.012		

Table S6. Relative Warps scores (RWs) describing variation in body shape of male and female mosquitofish separately, Related to Fig. 4. Singular values (SV), percentage explained for each relative warp (%), and cumulative percentage explained (Cum %) are shown.

Males				Females			
RW	SV	%	Cum %	RW	SV	%	Cum %
1	0.24	44.92	44.92	1	0.16989	26.54	26.54
2	0.15	17.15	62.07	2	0.14523	19.40	45.94
3	0.11	9.46	71.53	3	0.12591	14.58	60.52
4	0.09	6.76	78.30	4	0.11058	11.25	71.77
5	0.09	6.28	84.58	5	0.09395	8.12	79.89
6	0.08	4.72	89.30	6	0.08010	5.90	85.79
7	0.07	3.39	92.69	7	0.06186	3.52	89.31
8	0.05	2.05	94.74	8	0.05822	3.12	92.42
9	0.04	1.43	96.17	9	0.04502	1.86	94.29
10	0.04	1.11	97.28	10	0.04146	1.58	95.87
11	0.03	0.83	98.11	11	0.03707	1.26	97.13
12	0.03	0.66	98.77	12	0.03287	0.99	98.13
13	0.02	0.50	99.27	13	0.02826	0.73	98.86
14	0.02	0.35	99.62	14	0.02302	0.49	99.35
15	0.02	0.23	99.85	15	0.02018	0.37	99.72
16	0.01	0.15	100.00	16	0.01731	0.28	100.00

Table S7. Results from univariate linear-mixed models on group-behaviour measures before treatments started (i.e. week one), Related to Fig. 2a and Table 1. The dependent variables, social cohesion (AFND and AIID), activity (turning rate and distance swam), and space use (time in the central region), are tested with treatment (non-exposed and robot-exposed) and trial (two repeated measures per tank) as fixed effects, and mesocosm tank as the random effect (random intercepts). Analysis of variance was performed with Satterthwaite's method.

	Mosquitofish				Tadpoles				Mosquitofish & tadpoles			
Model	Mean sq.	*df*	*F*	*p*	Mean sq.	*df*	*F*	*p*	Mean sq.	*df*	*F*	*p*
AFND												
Treatment	1.489	1,10	0.603	0.455	0.363	1,10	0.160	0.698	3.701	1,10	0.952	0.352
Trial	4.366	1,11	1.769	0.210	3.066	1,11	1.350	0.270	1.138 10^{1}	1,11	2.928	0.115
AIID												
Treatment	0.366	1,10	0.277	0.610	0.176	1,10	0.161	0.697	1.277	1,10	0.636	0.444
Trial	0.429	1,11	0.324	0.580	1.494	1,11	1.365	0.267	2.785	1,11	1.386	0.264
Turning rate												
Treatment	0.044	1,10	2.851	0.122	0.195	1,10	1.257	0.288	-	-	-	-
Trial	0.071	1,11	4.544	0.056	0.007	1,11	0.047	0.833	-	-	-	-
Distance swam												
Treatment	3.189 10^{8}	1,10	1.939	0.194	1.913 10^{6}	1,10	0.022	0.885	-	-	-	-
Trial	1.626 10^{8}	1,11	0.989	0.341	9.608 10^{7}	1,11	1.096	0.317	-	-	-	-
Time in central region												
Treatment	6.180 10^{6}	1,10	0.842	0.380	2.601 10^{6}	1,10	0.544	0.478	-	-	-	-
Trial	2.627 10^{7}	1,11	3.580	0.085	6.799 10^{6}	1,11	1.422	0.258	-	-	-	-
